# Association of Cardiovascular Disease Risk Factor Burden With Progression of Coronary Atherosclerosis Assessed by Serial Coronary Computed Tomographic Angiography

**DOI:** 10.1001/jamanetworkopen.2020.11444

**Published:** 2020-07-24

**Authors:** Donghee Han, Daniel S. Berman, Robert J. H. Miller, Daniele Andreini, Matthew J. Budoff, Filippo Cademartiri, Kavitha Chinnaiyan, Jung Hyun Choi, Edoardo Conte, Hugo Marques, Pedro de Araújo Gonçalves, Ilan Gottlieb, Martin Hadamitzky, Jonathon Leipsic, Erica Maffei, Gianluca Pontone, Sangshoon Shin, Yong-Jin Kim, Byoung Kwon Lee, Eun Ju Chun, Ji Min Sung, Sang-Eun Lee, Renu Virmani, Habib Samady, Peter Stone, Jagat Narula, Jeroen J. Bax, Leslee J. Shaw, Fay Y. Lin, James K. Min, Hyuk-Jae Chang

**Affiliations:** 1 Department of Imaging and Medicine, Cedars-Sinai Medical Center, Los Angeles, California; 2 Division of Cardiology, Severance Cardiovascular Hospital, Yonsei University College of Medicine, Yonsei University Health System, Seoul, South Korea; 3 Department of Cardiac Sciences, University of Calgary, Calgary, Alberta, Canada; 4 Centro Cardiologico Monzino, Institute for Research, Hospitalization and Healthcare (IRCCS), Milan, Italy; 5 Department of Medicine, Los Angeles Biomedical Research Institute, Torrance, California; 6 Cardiovascular Imaging Center, SDN Institute, Institute for Research, Hospitalization and Healthcare (IRCCS), Naples, Italy; 7 Department of Cardiology, William Beaumont Hospital, Royal Oak, Michigan; 8 Pusan National University Hospital, Busan, South Korea; 9 UNICA, Unit of Cardiovascular Imaging, Hospital da Luz, Lisbon, Portugal; 10 Department of Radiology, Casa de Saúde São José, Rio de Janeiro, Brazil; 11 Department of Radiology and Nuclear Medicine, German Heart Center Munich, Munich, Germany; 12 Department of Medicine and Radiology, University of British Columbia, Vancouver, British Columbia, Canada; 13 Department of Radiology, Area Vasta 1–ASUR Marche, Urbino, Italy; 14 Ewha Womans University Seoul Hospital, Seoul, South Korea; 15 Seoul National University Hospital, Seoul, South Korea; 16 Gangnam Severance Hospital, Yonsei University College of Medicine, Seoul, South Korea; 17 Seoul National University Bundang Hospital, Sungnam, South Korea; 18 Department of Pathology, CVPath Institute, Gaithersburg, Maryland; 19 Division of Cardiology, Emory University School of Medicine, Atlanta, Georgia; 20 Cardiovascular Division, Harvard Medical School, Brigham and Women's Hospital, Boston, Massachusetts; 21 Zena and Michael A. Wiener Cardiovascular Institute, Icahn School of Medicine at Mount Sinai, Mount Sinai Heart, New York, New York; 22 Marie-Josée and Henry R. Kravis Center for Cardiovascular Health, New York, New York; 23 Department of Cardiology, Leiden University Medical Center, Leiden, the Netherlands; 24 Department of Radiology, New York–Presbyterian Hospital and Weill Cornell Medicine, New York, New York; 25 Cleerly, Inc, New York, New York

## Abstract

**Importance:**

Several studies have reported that the progression of coronary atherosclerosis, as measured by serial coronary computed tomographic (CT) angiography, is associated with the risk of future cardiovascular events. However, the cumulative consequences of multiple risk factors for plaque progression and the development of adverse plaque characteristics have not been well characterized.

**Objectives:**

To examine the association of cardiovascular risk factor burden, as assessed by atherosclerotic cardiovascular disease (ASCVD) risk score, with the progression of coronary atherosclerosis and the development of adverse plaque characteristics.

**Design, Setting, and Participants:**

This cohort study is a subgroup analysis of participant data from the prospective observational Progression of Atherosclerotic Plaque Determined by Computed Tomographic Angiography Imaging (PARADIGM) study, which evaluated the association between serial coronary CT angiography findings and clinical presentation. The PARADIGM international multicenter registry, which includes 13 centers in 7 countries (Brazil, Canada, Germany, Italy, Portugal, South Korea, and the US), was used to identify 1005 adult patients without known coronary artery disease who underwent serial coronary CT angiography scans (median interscan interval, 3.3 years; interquartile range [IQR], 2.6-4.8 years) between December 24, 2003, and December 16, 2015. Based on the 10-year ASCVD risk score, the cardiovascular risk factor burden was classified as low (<7.5%), intermediate (7.5%-20.0%), or high (>20.0%). Data were analyzed from February 8, 2019, to April 17, 2020.

**Exposures:**

Association of baseline ASCVD risk burden with plaque progression.

**Main Outcomes and Measures:**

Noncalcified plaque, calcified plaque, and total plaque volumes (mm^3^) were measured. Noncalcified plaque was subclassified using predefined Hounsfield unit thresholds for fibrous, fibrofatty, and low-attenuation plaque. The percent atheroma volume (PAV) was defined as plaque volume divided by vessel volume. Adverse plaque characteristics were defined as the presence of positive remodeling, low-attenuation plaque, or spotty calcification.

**Results:**

In total, 1005 patients (mean [SD] age, 60 [8] years; 575 men [57.2%]) were included in the analysis. Of those, 463 patients (46.1%) had a low 10-year ASCVD risk score (low-risk group), 373 patients (37.1%) had an intermediate ASCVD risk score (intermediate-risk group), and 169 patients (16.8%) had a high ASCVD risk score (high-risk group). The annualized progression rate of PAV for total plaque, calcified plaque, and noncalcified plaque was associated with increasing ASCVD risk (*r* = 0.26 for total plaque, *r* = 0.23 for calcified plaque, and *r* = 0.11 for noncalcified plaque; *P* < .001). The annualized PAV progression of total plaque, calcified plaque, and noncalcified plaque was significantly greater in the high-risk group compared with the low-risk and intermediate-risk groups (for total plaque, 0.99% vs 0.45% and 0.58%, respectively; *P* < .001; for calcified plaque, 0.61% vs 0.23% and 0.36%; *P* < .001; and for noncalcified plaque, 0.38%vs 0.22% and 0.23%; *P* = .01). When further subclassified by noncalcified plaque type, the annualized PAV progression of fibrofatty and low-attenuation plaque was greater in the high-risk group (0.09% and 0.02%, respectively) compared with the low- to intermediate-risk group (n = 836; 0.02% [*P* = .02] and 0.001% [*P* = .008], respectively). The interval development of adverse plaque characteristics was greater in the high-risk group compared with the low-risk and intermediate-risk groups (for new positive remodeling, 73 patients [43.2%] vs 151 patients [32.6%] and 133 patients [35.7%], respectively; *P* = .02; for new low-attenuation plaque, 26 patients [15.4%] vs 44 patients [9.5%] and 35 patients [9.4%]; *P* = .02; and for new spotty calcification, 37 patients [21.9%] vs 52 patients [11.2%] and 54 patients [14.5%]; *P* = .002). The progression of noncalcified plaque subclasses and the interval development of adverse plaque characteristics did not significantly differ between the low-risk and intermediate-risk groups.

**Conclusions and Relevance:**

Progression of coronary atherosclerosis occurred across all ASCVD risk groups and was associated with an increase in 10-year ASCVD risk. The progression of fibrofatty and low-attenuation plaques and the development of adverse plaque characteristics was greater in patients with a high risk of ASCVD.

## Key Points

**Question:**

Is the risk factor burden of cardiovascular disease, as assessed by atherosclerotic cardiovascular disease risk score, associated with coronary plaque progression and the development of adverse plaque characteristics?

**Findings:**

In this cohort study of 1005 adult patients from an international multicenter registry who underwent serial coronary computed tomographic angiography, the progression of coronary atherosclerotic plaque volume and the development of adverse plaque characteristics was greater in patients with a high atherosclerotic cardiovascular disease risk score.

**Meaning:**

The study findings suggest that the overall cardiovascular disease risk burden is associated with the progression of coronary atherosclerosis; the progression of fibrofatty plaque and low-attenuation plaque and the development of adverse plaque characteristics appear to be accelerated in patients with a high risk of atherosclerotic cardiovascular disease.

## Teaser

This cohort study analyzes data from the Progression of Atherosclerotic Plaque Determined by Computed Tomographic Angiography Imaging study to explore the association of cardiovascular risk factor burden with progression of coronary atherosclerosis and development of adverse plaque characteristics.

## Introduction

Coronary computed tomographic (CT) angiography allows quantitative measurement of multiple components of coronary atherosclerotic plaque and assessment of adverse plaque characteristics.^1,2,3,4^ In addition, the development and progression of coronary atherosclerotic plaque across the entire coronary tree can be evaluated using serial coronary CT angiography scans.^5,6^ Serial assessment of coronary artery plaques through coronary CT angiography provides clinical information regarding the progression of disease and the risk of experiencing future adverse cardiovascular events.^7,8^

Although several studies have reported an association between individual cardiovascular disease (CVD) risk factors and plaque progression,^9,10,11,12^ the cumulative consequences of multiple risk factors for plaque progression and the development of adverse plaque characteristics have not been well characterized. Current guidelines recommend the application of the 10-year atherosclerotic CVD (ASCVD) risk score,^13^ a validated model that incorporates age, sex, and traditional CVD risk factors to estimate the likelihood of cardiovascular events over 10 years.^14^ We aimed to explore the association of CVD risk factor burden, as measured by the 10-year ASCVD risk score, with coronary plaque progression and the development of adverse plaque characteristics in a large international longitudinal cohort using serial coronary CT angiography.

## Methods

### Study Population

The study population was acquired using data from the Progression of Atherosclerotic Plaque Determined by Computed Tomographic Angiography Imaging (PARADIGM) study. The PARADIGM registry has been previously described.^15^ In brief, the registry is a prospective international multicenter dynamic observational database designed to evaluate the association between serial coronary CT angiography findings and clinical presentation. Baseline data for the PARADIGM registry represent adult participants who received serial coronary CT angiography scans between December 24, 2003, and December 16, 2015, with follow-up through November 24, 2016. A total of 2252 consecutive adult participants underwent serial coronary CT angiography scans at an interval of 2 or more years at 1 of 13 centers in 7 countries (Brazil, Canada, Germany, Italy, Portugal, South Korea, and the US). Participating medical centers included the Montreal Heart Institute (Montreal, Quebec, Canada); Hospital da Luz (Lisbon, Portugal); the University of California, Los Angeles; the Institute for Research, Hospitalization and Healthcare (IRCCS; Milan, Italy), St.Paul’s Hospital (Vancouver, British Columbia, Canada); the University of Munich (Munich, Germany); Casa de Saúde (São José, Brazil); Severance Cardiovascular Hospital (Seoul, South Korea); Pusan University Hospital (Busan, South Korea); Seoul National University Hospital (Seoul, South Korea); Gangnam Severance Hospital (Seoul, South Korea); Seoul National University Bundang Hospital (Sungnam, South Korea); and Inje University Ilsan Paik Hospital (Goyang, South Korea). The study protocol was approved by the institutional review boards at all participating sites, and patients provided written informed consent. This study followed the Strengthening the Reporting of Observational Studies in Epidemiology (STROBE) reporting guideline for cohort studies.

Among 2252 consecutive participants, we excluded 754 patients with coronary CT angiography images that were inadequate for quantitative plaque analysis of the entire coronary tree, 282 patients who had a previous coronary revascularization, 133 patients who experienced an adverse cardiovascular event (defined as a myocardial infarction or revascularization) between serial coronary CT angiography scans, and 78 patients for whom the ASCVD risk score could not be calculated. After exclusions, 1005 patients were included in the current analysis. Baseline demographic characteristics, including age, sex, smoking status, and presence of hypertension, diabetes, or dyslipidemia, were collected at the baseline and follow-up coronary CT angiography scans. The 10-year ASCVD risk score, which was calculated using the pooled cohort equation^13^ based on information obtained at baseline coronary CT angiography, was used to assess CVD risk factor burden. Participants’ risk factor burdens were categorized as low (<7.5%), intermediate (7.5%-20.0%), or high (>20.0%).^14^

### Coronary CT Angiography

All testing, data acquisition, and image postprocessing for coronary CT angiography were performed in accordance with the Society of Cardiovascular Computed Tomography guidelines.^16^ The coronary CT angiography scans were acquired in all centers using a scanner with 64 or more detector rows. Baseline and follow-up data sets from each center were transferred to an offline workstation for analysis using semiautomated plaque analysis software (QAngio CT Research Edition, version 2.1.9.1; Medis Medical Imaging Systems) with manual correction as needed. Independent blinded readers who were experienced with coronary CT angiography (Core Cardiovascular Training Statement [COCATS] level 3 certification) analyzed all images. Segments were matched between baseline and follow-up coronary CT angiography scans using branch points as landmarks. For longitudinal comparisons of coronary CT angiography images, both baseline and follow-up coronary segments were coregistered using fiduciary landmarks, including distance from ostia or branch vessel takeoffs.

Plaques were qualitatively assessed for adverse characteristics, including positive remodeling, low-attenuation plaque, or spotty calcification. A remodeling index was defined as the maximal lesion vessel diameter divided by the proximal reference vessel diameter. Positive remodeling was defined as a remodeling index greater than 1.1, and low-attenuation plaque was defined as any voxel less than 30 Hounsfield units (HUs) within an individual coronary plaque.^7,17^ An intralesion calcific plaque less than 3 mm in length that composed less than 90 degrees of the lesion circumference was defined as spotty calcification.^17^ Development of adverse plaque characteristics was defined as the presence of a new lesion with adverse plaque characteristics on the follow-up coronary CT angiography scan or the development of adverse plaque characteristics from a lesion without adverse plaque characteristics on the baseline coronary CT angiography scan.

Plaque volumes (measured in mm^3^) of all coronary segments were obtained and summed to generate the total plaque volume on a per-patient level. Atherosclerotic plaque volume was further subclassified by composition, employing predefined intensity cutoff values in HU, including low-attenuation plaque (−30 to 30 HU), fibrofatty plaque (31-130 HU), fibrous plaque (131-350 HU), and calcified plaque (>350 HU).^18,19^ Percent atheroma volume (PAV) was defined as total plaque volume divided by vessel volume.^2^ The PAV was also calculated for each subtype of plaque composition. Rapid plaque progression was defined as an increase from baseline total PAV of more than 0.59% per year (the mean value of total PAV progression in the study population) on the follow-up coronary CT angiography scan.

### Statistical Analysis

Continuous variables were reported as mean (SD) or median (interquartile range [IQR]) and were compared using a *t* test or a Wilcoxon rank sum test (as appropriate) for 2-group comparisons and 1-way analysis of variance or a Kruskal-Wallis test (as appropriate) for comparisons of more than 2 groups. Categorical variables were reported as numbers and percentages and compared using the Pearson χ^2^ test. The association between ASCVD risk score and plaque progression was assessed using linear regression analyses and reported as correlation coefficient (β). All statistical tests were 2-sided and performed on independent or unpaired groups, with *P* < .05 considered statistically significant. All statistical analyses were performed using Stata software, version 13 (StataCorp LLC). Data were analyzed from February 8, 2019, to April 17, 2020.

## Results

### Baseline Characteristics

In total, 1005 patients (mean [SD] age, 60 [8] years; 575 men [57.2%]) were included in the analysis. The mean (SD) 10-year ASCVD risk score was 11.3 (9.9). The baseline characteristics of the study population according to 10-year ASCVD risk groups are shown in Table 1. A total of 463 patients (46.1%) had low 10-year ASCVD risk scores (low-risk group), 373 patients (37.1%) had intermediate 10-year ASCVD risk scores (intermediate-risk group), and 169 patients (16.8%) had high 10-year ASCVD risk scores (high-risk group). Patients with high ASCVD risk scores were older (mean [SD] age, 69 [6] years) compared with patients with low risk scores (mean [SD] age, 54 [6] years) and intermediate risk scores (mean [SD] age, 63 [6] years); and a greater proportion of patients with high ASCVD risk scores was male (124 patients [73.4%] vs 204 patients [44.1%] with low risk scores and 247 patients [66.2%] with intermediate-risk scores). Atypical chest pain was present in 694 patients (69.1%) at the baseline coronary CT angiography scan, and clinical symptom status did not significantly differ across ASCVD risk groups (Table 1). The prevalence of hypertension (115 patients [68.0%] in the high-risk group vs 188 patients [40.6%] in the low-risk group and 202 patients [54.2%] in the intermediate-risk group; *P* < .001), diabetes (84 patients [49.7%] in the high-risk group vs 45 patients [9.7%] in the low-risk group and 69 patients [18.5%] in the intermediate group; *P* < .001), and current smoking (54 patients [32.0%] in the high-risk group vs 46 patients [9.9%] in the low-risk group and 81 patients [21.7%] in the intermediate-risk group; *P* < .001) was greater in patients with higher 10-year ASCVD risk scores (Table 1).

**Table 1.** Baseline Characteristics Based on 10-Year Atherosclerotic Cardiovascular Disease Risk Score

Characteristic	No. (%)				*P* value
	All	Low risk	Intermediate risk	High risk	
Total No.	1005	463	373	169	
Age, mean (SD), y	60 (8)	54 (6)	63 (6)	69 (6)	<.001
Male sex	575 (57.2)	204 (44.1)	247 (66.2)	124 (73.4)	<.001
BMI, mean (SD)	25.3 (3.1)	24.9 (2.9)	25.5 (3.1)	25.7 (3.3)	.009
Clinical symptoms					.26
Asymptomatic	135 (13.4)	53 (11.4)	53 (14.2)	29 (17.2)	.15
Shortness of breath	53 (5.3)	16 (3.5)	25 (6.7)	12 (7.1)	.06
Atypical chest pain	694 (69.1)	332 (71.7)	249 (66.8)	113 (66.9)	.24
Noncardiac chest pain	80 (8.0)	41 (8.9)	31 (8.3)	8 (4.7)	.26
Typical chest pain	37 (3.7)	18 (3.9)	13 (3.5)	6 (3.6)	.95
Hypertension	505 (50.2)	188 (40.6)	202 (54.2)	115 (68.0)	<.001
Diabetes	198 (19.7)	45 (9.7)	69 (18.5)	84 (49.7)	<.001
Dyslipidemia	396 (39.4)	162 (35.0)	151 (40.5)	63 (37.3)	.24
Current smoker	181 (18.0)	46 (9.9)	81 (21.7)	54 (32.0)	<.001
Medications at baseline					
Aspirin	350 (34.8)	117 (25.2)	153 (41.0)	80 (47.3)	<.001
β-Blocker	232 (23.1)	64 (13.8)	113 (30.3)	55 (32.5)	<.001
ACE inhibitor/ARB	266 (26.5)	93 (20.1)	102 (27.3)	71 (42.0)	<.001
Statin use	363 (36.1)	136 (29.4)	156 (41.8)	71 (42.0)	<.001
ASCVD risk score	11.3 (9.9)	3.9 (1.9)	12.6 (3.5)	28.7 (9.6)	<.001

Abbreviations: ACE, angiotensin-converting enzyme; ARB, angiotensin II receptor blocker; ASCVD, atherosclerotic cardiovascular disease; BMI, body mass index (calculated as weight in kilograms divided by height in meters squared).

Coronary artery plaques were present in 760 patients (75.6%) at the baseline coronary CT angiography scan (Table 2). At the baseline scan, 300 patients (64.8%) in the low-risk group, 307 patients (82.3%) in the intermediate-risk group, and 153 patients (90.5%) in the high-risk group had coronary artery plaques (*P* < .001). Quantitative measurements of each type of plaque volume were significantly greater in patients with high 10-year ASCVD risk scores. For example, the median total plaque volume was 105.1 mm^3^ (IQR, 47.6-228.9 mm^3^) in the high-risk group compared with 23 mm^3^ (IQR, 0-85.9 mm^3^) in the low-risk group and 45.8 mm^3^ (IQR, 11.1-128.0 mm^3^) in the intermediate-risk group (*P* < .001) (Table 2).

**Table 2.** Baseline Coronary Computed Tomographic Angiography Measures Based on 10-Year Atherosclerotic Cardiovascular Disease Risk Score

Measure	No. (%)				
	All	Low risk	Intermediate risk	High risk	*P* value
Total No.	1005	463	373	169	
Presence of any plaque	760 (75.6)	300 (64.8)	307 (82.3)	153 (90.5)	<.001
Plaque volume, median (IQR), mm^3^					
Total plaque	43.1 (3.9-127.0)	23 (0-85.9)	45.8 (11.1-128.0)	105.1 (47.6-228.9)	<.001
Calcified plaque	5.3 (0-32.6)	1.2 (0-16.4)	7.8 (0.1-37.2)	31.3 (3.8-75.3)	<.001
Noncalcified plaque	28.8 (1.0-85.9)	14.8 (0-61.4)	30.5 (5.9-89.4)	66.3 (23.8-149.2)	<.001
Fibrous plaque	20.3 (0.7-56.5)	10.3 (0-41.6)	22.3 (4.6-55.8)	46.3 (20.7-96.3)	<.001
Fibrofatty plaque	3.5 (0-22.1)	1.0 (0-17.3)	4.6 (0.1-19.6)	11.7 (1.8-34.6)	<.001
Low-attenuation plaque	0 (0-1.5)	0 (0-0.8)	0 (0-1.6)	0.3 (0-2.8)	<.001
Adverse plaque characteristics					
Any APC	688 (68.5)	260 (56.2)	280 (75.1)	148 (87.6)	<.001
Positive remodeling	662 (65.9)	250 (54.0)	268 (71.8)	144 (85.2)	<.001
Low-attenuation plaque	213 (21.2)	88 (19.0)	76 (20.4)	49 (29.0)	.02
Spotty calcification	194 (19.3)	68 (14.7)	78 (20.9)	48 (28.4)	<.001

Abbreviations: APC, adverse plaque characteristic; IQR, interquartile range.

The prevalence of any adverse plaque characteristics at the baseline coronary CT angiography scan was 260 patients (56.2%) in the low-risk group, 280 patients (75.1%) in the intermediate-risk group, and 148 patients (87.6%) in the high-risk group (*P* < .001) (Table 2). The prevalence of positive remodeling, low-attenuation plaque, and spotty calcification was significantly higher in patients with a high ASCVD risk score (144 patients [85.2%], 49 patients [29.0%], and 48 patients [28.4%], respectively) compared with patients with a low ASCVD risk score (250 patients [54.0%], 88 patients [10.0%], and 68 patients [14.7%], respectively) or an intermediate ASCVD risk score (268 patients [71.9%], 76 patients [20.4%], and 78 patients [20.9%], respectively; *P* < .001 for positive remodeling, *P* = .02 for low-attenuation plaque, and *P* < .001 for spotty calcification).

### Plaque Progression and ASCVD Risk

The median interval between coronary CT angiography scans was 3.3 years (IQR, 2.6-4.8 years), with no significant difference in interval across ASCVD risk groups (median, 3.3 years [IQR, 2.5-4.8 years] for the low-risk group, 3.4 years [IQR, 2.6-4.8 years] for the intermediate-risk group, and 3.4 years [IQR, 2.7-4.9 years] for the high-risk group; *P* = .57). The annual progression of PAV for total plaque, calcified plaque, and noncalcified plaque was weakly correlated with ASCVD risk (*r* = 0.26 for total plaque, 0.23 for calcified plaque, and 0.11 for noncalcified plaque; *P* < .001) (eFigure in the Supplement). The annualized progression of total plaque, calcified plaque, and noncalcified plaque was significantly greater in the high-risk group compared with the low-risk and intermediate-risk groups (for total plaque, 0.99% vs 0.45% and 0.58%, respectively; *P* < .001; for calcified plaque, 0.61% vs 0.23% and 0.36%; *P* < .001; and for noncalcified plaque, 0.38% vs 0.22% and 0.23%; *P* = .01). The results of linear regression analyses for the association between the annualized PAV progression of total plaque and the ASCVD risk score are shown in Table 3. In the multivariate analysis, the ASCVD risk score was significantly correlated with the annualized PAV progression of total plaque (β = 0.108; SE = 0.238; *P* < .001) after adjusting for statin use, PAV, and the presence of adverse plaque characteristics at the baseline coronary CT angiography scan.

**Table 3.** Linear Regression Analysis for the Association of Clinical and Plaque Characteristics With Plaque Progression

Variable	Univariate		Multivariate	
	β *(*SE)	*P* value	β (SE)	*P* value
Statin use	0.139 (0.056)	<.001	0.012 (0.046)	.67
Diameter stenosis	0.302 (0.001)	<.001	0.006 (0.133)	.84
Baseline PAV	0.540 (0.409)	<.001	0.445 (0.509)	<.001
Positive remodeling	0.347 (0.054)	<.001	0.105 (0.055)	.001
Low-attenuation plaque	0.228 (0.065)	<.001	0.044 (0.059)	.12
Spotty calcification	0.209 (0.067)	<.001	0.005 (0.061)	.86
ASCVD risk score	0.259 (0.264)	<.001	0.108 (0.238)	<.001

Abbreviations: ASCVD, atherosclerotic cardiovascular disease; PAV, percent atheroma volume.

**Figure 1.**
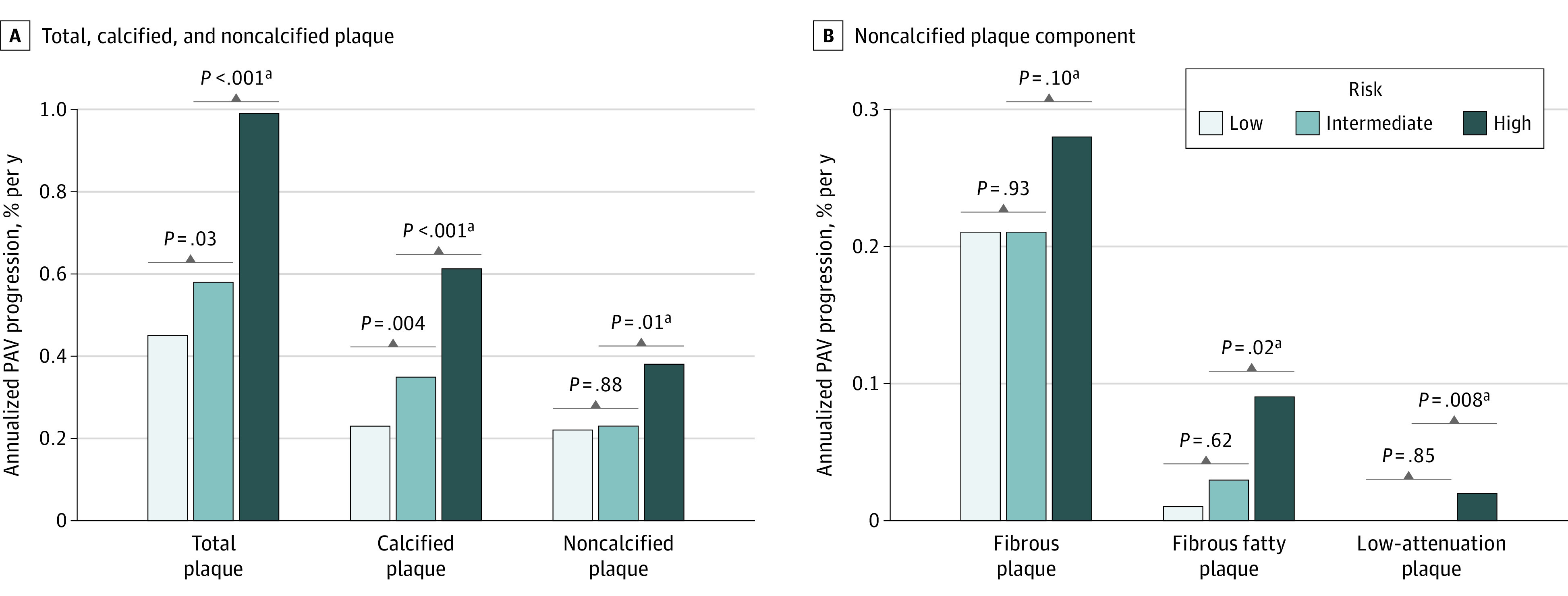
Annualized Plaque Progression A, Total, calcified, and noncalcified plaque. B, Noncalcified plaque component. Plaque progression according to atherosclerotic cardiovascular disease risk score. PAV indicates percent atheroma volume. ^a^Comparison between high-risk group vs low- to intermediate-risk group (n = 836).

The annualized PAV progression according to plaque components is described in Figure 1 and eTable 1 in the Supplement. The annualized progression of PAV for total plaque and calcified plaque increased as ASCVD risk increased (Figure 1A). The annualized PAV progression of noncalcified plaque was significantly greater in the high-risk group (0.38%) compared with the low- to intermediate-risk group (n = 836; 0.22%; *P* = .01) (Figure 1A). When we further subclassified noncalcified plaque, the annualized PAV progression of fibrofatty plaque and low-attenuation plaque was significantly greater in the high-risk group (0.09% and 0.02%, respectively) compared with the low- to intermediate-risk group (n = 836; 0.02%; *P* = .02 and 0.001%; *P* = .008, respectively) (Figure 1B). No significant differences between the low-risk and intermediate-risk groups were found in the progression of annualized PAV for noncalcified plaque subclasses.

**Figure 2.**
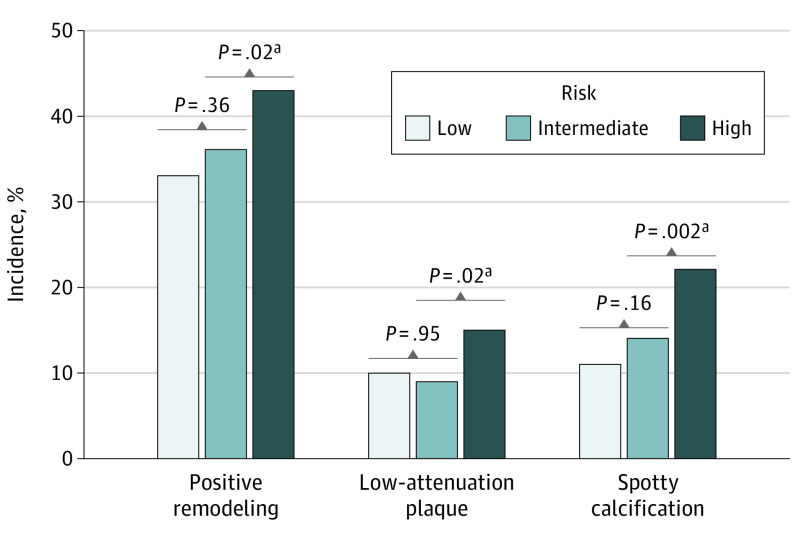
Newly Developed Adverse Plaque Characteristics in Follow-up Coronary Computed Tomographic Angiography Adverse plaque characteristics according to atherosclerotic cardiovascular disease risk groups. ^a^Comparison between high-risk group vs low- to intermediate-risk group (n = 836) and intermediate-risk group to low-risk group.

The incidence of new adverse plaque characteristics in the follow-up coronary CT angiography scan according to ASCVD risk groups are described in Figure 2 and eTable 1 in the Supplement. The development of new positive remodeling, new low-attenuation plaque, and new spotty calcification on the follow-up coronary CT angiography scan was greater in the high-risk group (73 patients [43.2%], 26 patients [15.4%], and 37 patients [21.9%] of 169 total patients, respectively) compared with the low-risk group (151 patients [32.6%], 44 patients [9.5%], and 52 patients [11.2%] of 463 total patients, respectively) and the intermediate-risk group (133 patients [35.7%], 35 patients [9.4%], and 54 patients [14.5%] of 373 total patients, respectively; *P* = .02 for new positive remodeling, *P* = .02 for new low-attenuation plaque, and *P* = .002 for new spotty calcification) (Figure 2). The incidence of adverse plaque characteristics did not significantly differ between the low-risk and intermediate-risk groups.

### Rapid Plaque Progression

A total of 77 of 169 patients (45.6%) with a high risk of ASCVD experienced rapid plaque progression. Patients with high ASCVD risk and rapid plaque progression were more frequently men (50 of 77 patients [64.9%]) and had more clinical risk factors (59 patients [76.6%] had hypertension, 48 patients [62.3%] had diabetes, and 32 patients [41.6%] were current smokers) compared with those without rapid plaque progression (27 of 92 patients were men [29.3%]; *P* = .02; 56 patients [60.9%] had hypertension [*P* = .04], 36 patients [39.1%] had diabetes [*P* = .003], and 22 patients [23.9%] were current smokers [*P* = .01]) (eTable 2 in the Supplement). However, age and ASCVD risk score did not differ between patients with rapid progression compared with those without rapid progression (mean [SD] age, 69 [5] years vs 69 [6] years, respectively; *P* = .99; mean [SD] ASCVD risk score, 29.9 [10.6] vs 27.5 [8.5], respectively; *P* = .10).

Quantitative measurements of each type of plaque volume were significantly higher in patients with rapid progression (median volume for total plaque, 185.7 mm^3^ [IQR, 93.9-367.7 mm^3^]; median volume for calcified plaque, 56.7 mm^3^ [IQR, 23.4-129.2 mm^3^]; median volume for noncalcified plaque, 118.5 mm^3^ [IQR, 52.7-228.4 mm^3^]) compared with patients without rapid progression (median volume for total plaque, 57.8 mm^3^ [IQR, 20.5-120.3 mm^3^]; median volume for calcified plaque, 13.6 mm^3^ [IQR, 0-43.3 mm^3^]; median volume for noncalcified plaque, 38.9 mm^3^ [IQR, 10.4-85.3 mm^3^]; *P* < .001 for all comparisons). The prevalence of positive remodeling and low-attenuation plaque at the baseline coronary CT angiography scan was also significantly higher in patients with rapid progression (74 patients [96.1%] and 30 patients [39.0%], respectively) compared with those without rapid progression (70 patients [76.1%] and 19 patients [20.7%], respectively; *P* < .001 for positive remodeling and *P* = .009 for low-attenuation plaque) (eTable 2 in the Supplement). Notably, all patients with a high risk of ASCVD and rapid plaque progression had coronary plaques at the baseline coronary CT angiography scan.

Among 2252 patients in the PARADIGM registry, 754 patients were excluded due to image quality. Compared with the 1005 patients in the analysis group, the excluded patients were older (mean [SD] age, 60 [8] years vs 63 [10] years, respectively; *P* < .001), more frequently male (575 men [57.2%] vs 502 men [66.6%]; *P* < .001), and had more clinical risk factors (505 patients [50.3%] vs 485 patients [64.3%] had hypertension [*P* < .001], 198 patients [19.7%] vs 208 patients [27.6%] had diabetes [*P* < .001], and 376 patients [39.4%] vs 360 patients [47.8%] had dyslipidemia [*P* < .001]) (eTable 3 in the Supplement).

## Discussion

In this study, we found that overall CVD risk burden was associated with the progression of coronary atherosclerosis. A high risk of ASCVD was associated with more rapid progression of coronary atherosclerosis, including calcified plaque, fibrofatty plaque, and low-attenuation plaque volumes, on serial coronary CT angiography scans as measured by the PAV. Furthermore, the incidence of new adverse plaque characteristics increased among patients who had a high risk of ASCVD compared with those who had a low to intermediate risk.

Previous studies of patients who underwent serial coronary CT angiography examinations have reported that clinical factors and laboratory values are associated with the rate of plaque progression.^10,11,12^ The presence of conventional risk factors, such as diabetes^9,10^ or high low-density lipoprotein cholesterol levels,^11,12^ are associated with accelerated plaque progression. Our findings expand on these earlier findings by indicating that patients with a higher CVD risk factor burden, assessed with a validated global risk score, have accelerated plaque progression compared with patients with a lower risk. Most patients with CVD do not present with a single CVD risk factor,^20,21^ and physicians typically integrate multiple conventional CVD risk factors in clinical practice.^14,22^ Therefore, the current findings provide practical insights on the development and evolution of atherosclerotic plaques based on a patient’s overall CVD risk burden as assessed by the ASCVD risk score.

A growing body of evidence suggests that noncalcified plaque components are more closely associated with CVD risk than calcified plaque components. The 2018 Incident Coronary Syndromes Identified by Computed Tomography (ICONIC) study observed that patients with acute coronary syndrome associated with coronary plaques had substantially larger noncalcified plaque volumes, most notably low-attenuation plaque volume, before they developed acute coronary syndrome compared with a similar degree of stenotic plaque in patients without acute coronary syndrome, while there was no difference in total plaque and calcified plaque volumes.^21^ In the current study, we found that although plaque progression occurred in patients with low to intermediate risk, the progression of high-risk noncalcified plaque components, including fibrofatty plaque and low-attenuation plaque volumes, was significantly accelerated in patients with a high CVD risk factor burden compared with patients with a low to intermediate risk factor burden. However, it is unknown whether interventions in these patients were associated with improvements in clinical outcomes.

Because coronary atherosclerosis is a dynamic process, with plaques gaining or losing adverse plaque characteristics over time, the development of adverse plaque characteristics may be an important step from subclinical atherosclerosis to an acute coronary syndrome event.^7,23^ In a study of 449 patients who underwent serial coronary CT angiography scans, the development of high-risk plaque features (ie, positive remodeling and low-attenuation plaque) was independently associated with acute coronary syndrome.^7^ In the current study, we observed that a high CVD risk factor burden indicated not only a high prevalence of adverse plaque characteristics at the baseline coronary CT angiography scan but also a significant acceleration in the development of new adverse plaque characteristics.

Although plaque progression has been reported to be associated with an increased risk of developing CVD, methods and thresholds to assess clinically significant plaque progression were inconsistent in previous investigations. Using serial intravascular ultrasound, Nicholls et al^24^ reported a prognostic significance of per-lesion–based plaque progression at an annualized PAV of more than 1%. With regard to coronary CT angiography, Motoyama et al^7^ reported prognostic significance of plaque progression for acute coronary syndrome, in which progression was defined as an increase in the degree of stenosis or a remodeling index of more than 1.1. In the current study, the rapid plaque progression was defined as an increase from the baseline total PAV of more than the mean value of total PAV progression in the study population because there is no established threshold for significant plaque progression as assessed by volumetric computed tomographic plaque quantification of the entire coronary tree. Future studies are warranted to establish a clinically relevant threshold of total coronary plaque progression by serial coronary CT angiography assessment.

The current study results indicated that up to two-thirds of low-risk patients (ie, those with <7.5%) had coronary artery plaques at the baseline coronary CT angiography scan. In addition, the progression of noncalcified plaque components and the incidence of high-risk plaque were only modestly different between the low and intermediate ASCVD risk groups. These findings suggest that the arbitrary cutoffs of 5.0% to 7.5% (borderline) or 7.5% to 20.0% (intermediate) of the 10-year ASCVD score are limited in their ability to accurately differentiate the actual ASCVD risk among individuals in the low-risk to intermediate-risk populations. This finding is consistent with the current guideline, which emphasizes the need for risk-enhancing factors to reclassify ASCVD risk in the borderline to intermediate-risk group.^14^ Noninvasive coronary imaging, such as coronary calcium scans or coronary CT angiography scans, may help to personalize risk assessment and shared decision-making regarding the intensity needed for the preventive strategy.^25,26,27^

### Limitations

This study has several limitations. Although the PARADIGM registry is, to our knowledge, the largest serial coronary CT angiography study to date, there may be unmeasured confounding factors which have implications for the results of this study. We did not have detailed medication information or measures of patient adherence during the interval between scans, which could be used to perform a more refined analysis of the association of statin therapy with plaque progression. The ASCVD risk score was originally validated for 10-year outcomes in asymptomatic patients; therefore, the ASCVD risk score may not completely reflect risk in symptomatic patients who are referred for coronary CT angiography. We included patients who had coronary CT angiography scans with sufficient image quality to allow quantitative assessment of both the baseline and follow-up scans for the purpose of assessing plaque progression in the entire coronary tree. Compared with the excluded patients, the analyzed patients had a lower prevalence of cardiovascular risk factors and medication receipt (eTable 3 in the Supplement). Thus, the potential consequences of selection bias for the generalizability of the findings should be considered. Patients who underwent revascularization before their follow-up coronary CT angiography scan were excluded; thus, the study population consisted mainly of low-risk patients. As a result, the overall clinical event rate in our cohort is low, precluding us from exploring the potential associations between plaque progression, CVD risk factor burden, and adverse CVD outcomes.

## Conclusions

The progression of coronary atherosclerosis occurs across all ASCVD risk groups, but the progression of overall PAV increases as the 10-year ASCVD risk score increases. The progression of fibrofatty and low-attenuation plaques and the development of adverse plaque characteristics was greater in patients with a high risk of ASCVD.

## Notes

Supplement. **eTable 1.** Annualized PAV Progression and Development of New APC According to ASCVD Risk Score **eTable 2.** Baseline Characteristics of Patients With High ASCVD Risk According to Rapid Plaque Progression **eTable 3.** Difference Between Analyzed and Excluded Patients Due to Image Quality **eFigure.** Association Between ASCVD Risk Score and Annualized Plaque Progression
