# Supplementary material for: Association of Cardiovascular Disease Risk Factor Burden With Progression of Coronary Atherosclerosis Assessed by Serial Coronary Computed Tomographic Angiography
Source: JAMA Netw Open. 2020 Jul 24;3(7):e2011444. doi: 10.1001/jamanetworkopen.2020.11444 (PMC7382001; doi:10.1001/jamanetworkopen.2020.11444)
Supplement: Supplement. — eTable 1. Annualized PAV Progression and Development of New APC According to ASCVD Risk Score eTable 2. Baseline Characteristics of Patients With High ASCVD Risk According to Rapid Plaque Progression eTable 3. Difference Between Analyzed and Excluded Patients Due to Image Quality eFigure. Association Between ASCVD Risk Score and Annualized Plaque Progression [file jamanetwopen-3-e2011444-s001.pdf]

Supplementary Online Content

Han D, Berman DS, Miller RJH, et al. Association of cardiovascular disease risk factor burden with progression of coronary atherosclerosis assessed by serial coronary computed tomographic angiography. *JAMA Netw Open.* 2020;3(7):e2011444. doi:10.1001/jamanetworkopen.2020.11444

eTable 1. Annualized PAV Progression and Development of New APC According to ASCVD Risk Score

eTable 2. Baseline Characteristics of Patients With High ASCVD Risk According to Rapid Plaque Progression

eTable 3. Difference Between Analyzed and Excluded Patients Due to Image Quality

eFigure. Association Between ASCVD Risk Score and Annualized Plaque Progression

This supplementary material has been provided by the authors to give readers additional information about their work.

© 2020 Han D et al. *JAMA Network Open*.

eTable 1. Annualized PAV Progression and Development of New APC According to ASCVD Risk Score

	All	Low risk	Intermediate risk	High risk	*P* value for trend	*P* value for low vs. intermediate	*P* value for low to intermediate vs. high
Total plaque	0.592 ± 0.859	0.454 ± 0.734	0.584 ± 0.753	0.989 ± 1.207	<.001	.03	<.001
Calcified plaque	0.342 ± 0.616	0.233 ± 0.506	0.356 ± 0.611	0.607 ± 0.797	<.001	.004	<.001
Noncalcified plaque	0.250 ± 0.733	0.220 ± 0.662	0.227 ± 0.600	0.382 ± 1.089	<.001	.88	.01
Fibrous plaque	0.220 ± 0.524	0.209 ± 0.503	0.206 ± 0.442	0.280 ± 0.712	.26	.93	.10
Fibrofatty plaque	0.029 ± 0.349	0.013 ± 0.329	0.025 ± 0.285	0.085 ± 0.499	.07	.62	.02
Low-attenuation plaque	0.002 ± 0.008	0.000 ± 0.082	0.002 ± 0.08	0.017 ± 0.082	.03	.85	.008
New positive remodeling	357 (35.5)	151 (32.6)	133 (35.7)	73 (43.2)	.05	.36	.02
New low-attenuation plaque	105 (10.4)	44 (9.5)	35 (9.4)	26 (15.4)	.07	.95	.02
New spotty calcification	143 (14.2)	52 (11.2)	54 (14.5)	37 (21.9)	.003	.16	.002

Abbreviations: ASCVD, atherosclerotic cardiovascular disease; PAV, percent atheroma volume; APC, adverse plaque characteristics

Values are shown as mean ± standard deviation or number (percentage).

© 2020 Han D et al. *JAMA Network Open*.

eTable 2. Baseline Characteristics of Patients With High ASCVD Risk According to Rapid Plaque Progression

	No rapid progression (n = 92)	Rapid progression (n = 77)	*P* value
Age, y	69 ± 6	69±5.2	.99
Male	27 (29.3)	50 (64.9)	.02
Body mass index	25.7 ± 30	25.8±3.6	.76
Clinical symptoms			.24
Asymptomatic	21 (22.8)	8 (10.4)	.03
Shortness of breath	6 (6.5)	6 (7.8)	.75
Atypical chest pain	58 (63.0)	55 (71.4)	.25
Noncardiac chest pain	5 (5.4)	3 (3.9)	.64
Typical chest pain	2 (2.2)	4 (5.2)	.29
Hypertension	56 (60.9)	59 (76.6)	.04
Diabetes	36 (39.1)	48 (62.3)	.003
Dyslipidemia	35 (38.0)	28 (36.4)	.78
Current smoker	22 (23.9)	32 (41.6)	.01
ASCVD score	27.5 ± 8.5	29.9 ± 10.6	.10
Baseline CCTA characteristics			
Presence of any plaque	76 (82.6)	77 (100.0)	<.001
Plaque volume, mm^3			
Total plaque	57.8 (20.5-120.3)	185.7 (93.9-367.7)	<.001
Calcified plaque	13.6 (0-43.3)	56.7 (23.4-129.2)	<.001
Noncalcified plaque	38.9 (10.4-85.3)	118.5 (52.7-228.4)	<.001
Fibrous plaque	26.1 (7.2-60.4)	79.8 (40.2-156.3)	<.001
Fibrofatty plaque	5.3 (0.2-25.4)	19.5 (4.2-52.7)	<.001
Low-attenuation plaque	0.1 (0-1.9)	0.72 (0-5.4)	.01
APCs			
Any APCs	72 (78.3)	76 (98.7)	<.001
Positive remodeling	70 (76.1)	74 (96.1)	<.001
Low-attenuation plaque	19 (20.7)	30 (38.9)	.009
Spotty calcification	22 (23.9)	26 (33.8)	.12

Abbreviations: APC, adverse plaque characteristics; ASCVD, atherosclerotic cardiovascular disease; CCTA, coronary computed tomographic angiography.

Values are shown as mean ± standard deviation, median (interquartile range) or number (percentage).

© 2020 Han D et al. *JAMA Network Open*.

eTable 3. Difference Between Analyzed and Excluded Patients Due to Image Quality

	Analyzed (n = 1005)	Excluded due to image quality (n = 754)	*P* value
Age, y	60 ± 8	63 ± 10	<.001
Male	575 (57.2)	502 (66.6)	<.001
Body mass index	25.3 ± 3.1	25.6 ± 3.6	.02
Clinical symptoms			.26
Asymptomatic	135 (13.4)	287 (38.1)	<.001
Shortness of breath	53 (5.3)	70 (9.3)	.004
Atypical chest pain	694 (69.1)	348 (46.2)	<.001
Noncardiac chest pain	80 (7.9)	47 (6.2)	.04
Typical chest pain	37 (3.7)	45 (5.9)	.02
Hypertension	505 (50.3)	485 (64.3)	<.001
Diabetes	198 (19.7)	208 (27.6)	<.001
Dyslipidemia	396 (39.4)	360 (47.8)	<.001
Current smoker	181 (18.0)	150 (19.9)	.22
Medications at baseline			
Aspirin	350 (34.8)	405 (53.7)	<.001
Beta blocker	232 (23.1)	345 (45.8)	<.001
ACE inhibitor/ARB	266 (26.5)	296 (39.3)	<.001
Statin use	363 (36.2)	420 (55.7)	<.001

Abbreviations: ACE, angiotensin-converting enzyme; ARB, angiotensin II receptor blockers; ASCVD, atherosclerotic cardiovascular disease.

Values are shown as mean ± standard deviation or number (percentage).

© 2020 Han D et al. *JAMA Network Open*.

eFigure. Association Between ASCVD Risk Score and Annualized Plaque Progression

A) Total plaque

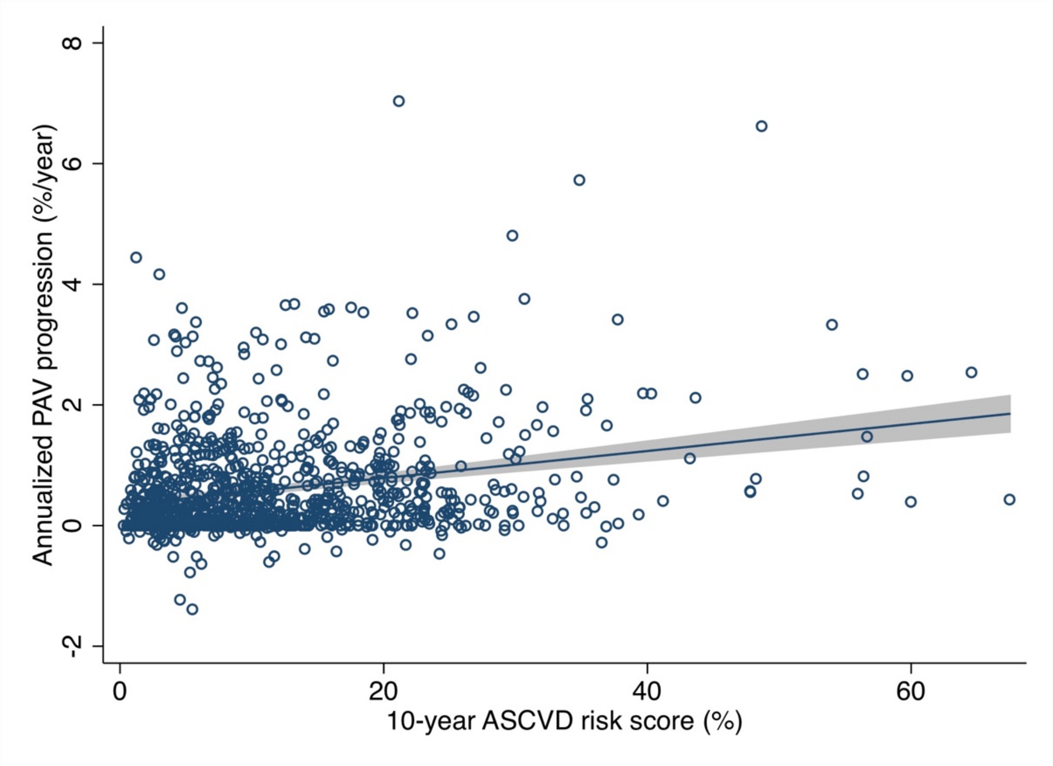

Abbreviation: ASCVD, atherosclerotic cardiovascular disease. PAV. Percent atheroma volume

© 2020 Han D et al. *JAMA Network Open*.

B) Calcified plaque

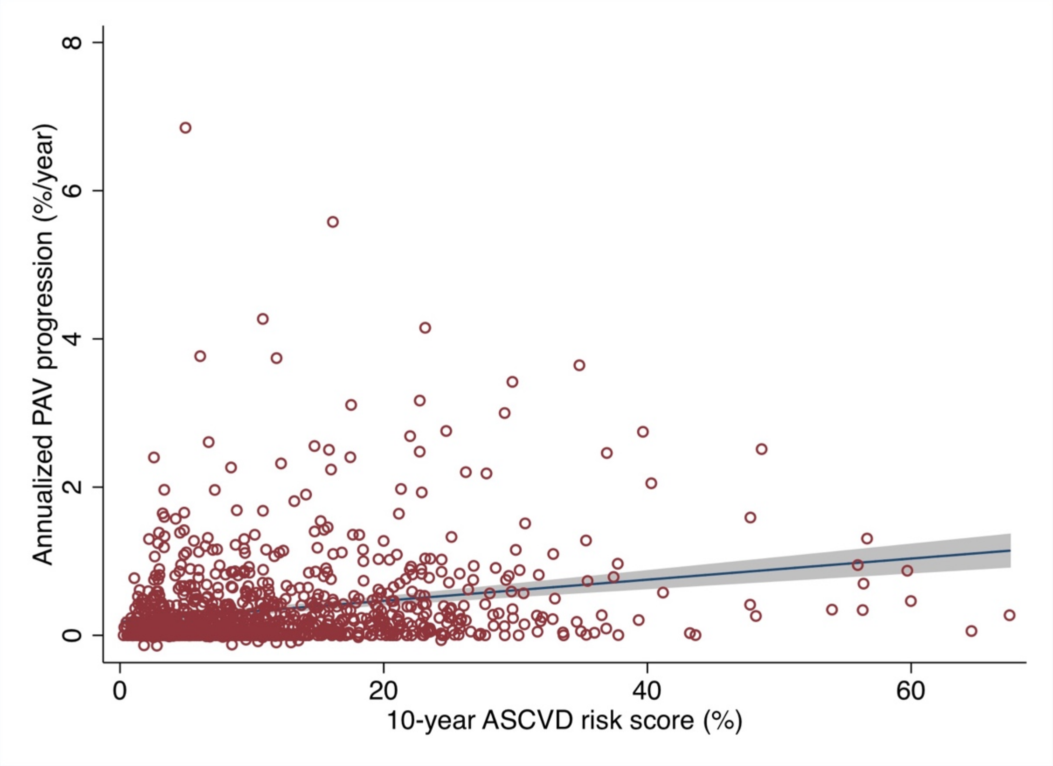

Abbreviation: ASCVD, atherosclerotic cardiovascular disease. PAV. Percent atheroma volume

C) Noncalcified plaque

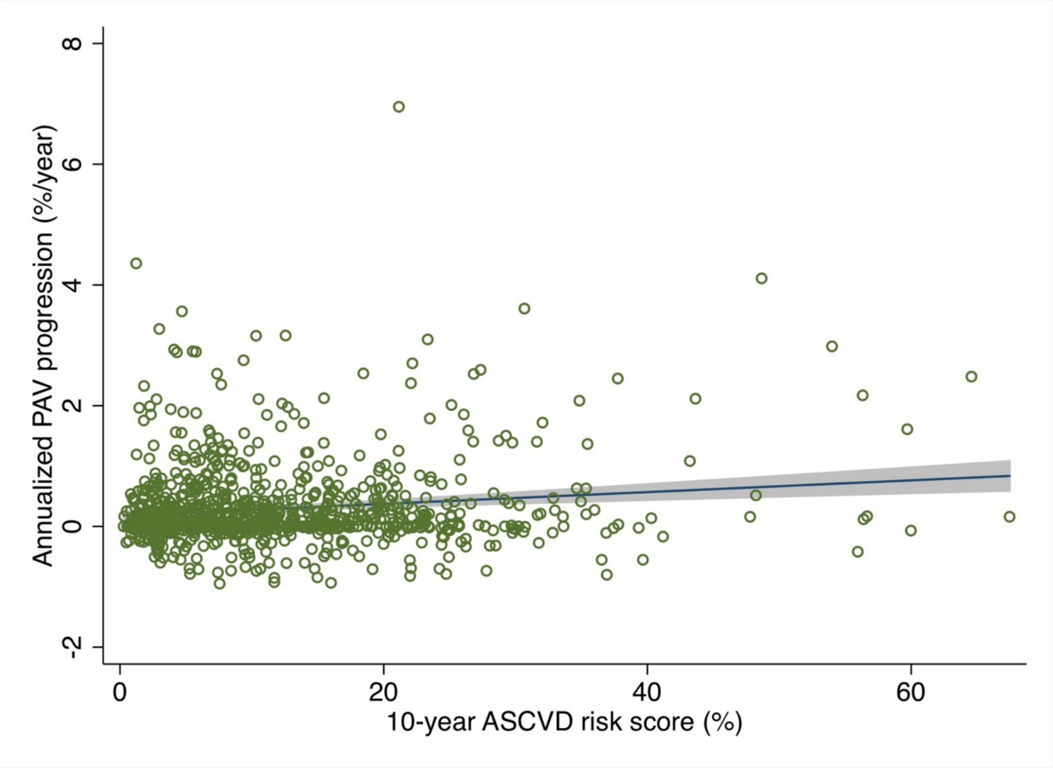

Abbreviation: ASCVD, atherosclerotic cardiovascular disease. PAV. Percent atheroma volume

© 2020 Han D et al. *JAMA Network Open*.
